# Assessing the Influence of Intermittent Alcohol Access on Acrylamide-Induced Neuronal Toxicity in an Experimental Rat Model

**DOI:** 10.3390/brainsci14060574

**Published:** 2024-06-04

**Authors:** Abdulaziz Arif A. Alshammari, Awyed Batah Almutairi, Minhajul Arfeen, Abdullah Saleh Alkhamiss, Maha A. Aldubayan, Ahmad H. Alhowail, Vasudevan Mani

**Affiliations:** 1 Department of Pharmacology and Toxicology, College of Pharmacy, Qassim University, Buraydah 51452, Saudi Arabia; aziz.alhmzani@gmail.com (A.A.A.A.);; 2 Pharmacy Care Department, Al Midhnab General Hospital, Qassim Health Cluster, Ministry of Health, Al Mithnab 56526, Saudi Arabia; 3 Department of Medicinal Chemistry and Pharmacognosy, College of Pharmacy, Qassim University, Buraydah 51452, Saudi Arabia; m.arfeen@qu.edu.sa; 4 Department of Pathology, College of Medicine, Qassim University, Buraydah 51452, Saudi Arabia

**Keywords:** acrylamide, ethanol, memory dysfunctions, neurotoxicity, oxidative stress, neuroinflammation, apoptosis

## Abstract

Tobacco and alcohol have been identified as health risk behaviors associated with significant unfavorable health consequences, ranking within the list of the top ten causes of mortality and disability-adjusted life years (DALY). The combustion of tobacco leads to the formation of acrylamide (ACR), which is well known for its neurotoxic effects. Similarly, alcohol consumption has also been widely recognized for its neurotoxic effects. Both substances can affect neurons and neuroglia cells through various pathways. This study sought to examine the impacts of co-administration of ACR and intermittent-access ethanol (IAE) consumption over a period of one month. The experimental group received 20 mg/kg of ACR, administered orally, along with IAE of 20% ethanol sessions lasting 24 h, three times per week. The cognitive outcomes were assessed utilizing the elevated plus maze (EPM), which was employed as a means of assessing the capability to learn and remember, the novel object recognition (NOR) test, which was employed to assess recognition memory, and the Y-maze, which was used to explore a new environment and navigate. Additionally, ELISA assays were performed to examine underlying mechanisms, including markers associated with inflammation (NF-κB, PGE2, and TNF-α), apoptosis (Bcl2, Bax, and Caspase-3), and oxidative stress (MDA, catalase, and GSH). These markers were assessed in the brain homogenate as part of the investigation. Furthermore, a histopathological study was conducted. The findings indicated that NF-κB levels increased significantly in the combination of ACR and IAE groups (ACR + IAE) compared to either the ACR-alone or IAE-alone groups. However, parallel changes were observed in TNF-α, PGE2, Bax, Bcl-2, Caspase-3, GSH, and CAT levels when comparing the ACR + IAE group to the ACR-alone group. Comparable alterations were noted between the ACR + IAE treatment and IAE-alone groups in TNF-α, Bcl-2, MDA, GSH, and CAT levels. Moreover, the histopathological analysis revealed significant changes between the ACR + IAE and the ACR- or IAE-alone groups. Regarding memory parameters assessed using tests including EPM, NOR, and Y-maze, considerable changes were observed across all treatment groups as opposed to the control. Surprisingly, there were no notable differences in the NOR and Y-maze tasks between the alone and combination treatment. Further study is necessary to explore the long-term alteration of co-administering ACR and IAE on behavior, memory, and neurotoxicity-related mechanisms, in order to elucidate their combined effects more clearly.

## 1. Introduction

Based on a 2023 report by the World Health Organization (WHO), the need to regulate and combat tobacco use remains a top priority in the global health agenda, as tobacco consumption continues to be a significant concern for public health worldwide. This concern is supported by the annual toll of over 8 million deaths caused by tobacco-related issues [1]. Every year, millions of individuals suffer illnesses and lose their lives due to the consumption of tobacco in various forms [2]. Many researchers have suggested that long-term cigarette smoking is linked to a higher likelihood of various medical conditions that can potentially impact brain neurobiology and neurocognition, either directly or indirectly [3,4]. In cross-sectional studies focusing on smokers who are middle-aged and/or in older adulthood, researchers have noted that smokers exhibit poorer cognitive performance in multiple areas compared to non-smoking control groups [5]. Another study indicated that smoking cigarettes may hasten the aging process of the brain [6]. Even as rates of tobacco use decline, the number of yearly fatalities is anticipated to continue increasing due to the gradual harm caused by tobacco to both its users and those exposed to its emissions [7].

**Figure 1:**
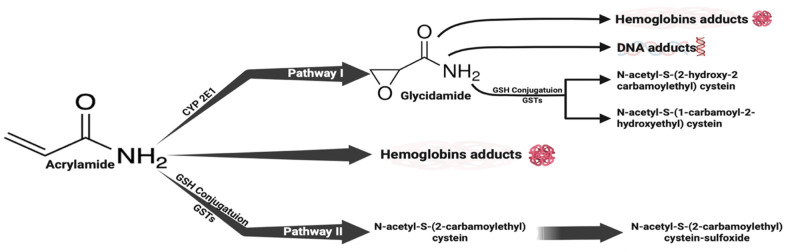
Acrylamide (ACR) is metabolized within the body through two primary pathways. The first pathway involves the enzymatic reaction catalyzed by CYP2E1, which converts ACR into its metabolite, glycidamide (GA). GA has been associated with various effects, such as the formation of DNA adducts, inhibition of neurotransmitter release, nerve terminal degeneration, and damage to nerve structures. Therefore, it is believed that the neurotoxicity induced by ACR primarily occurs through the actions of GA. The second pathway involves the biotransformation of ACR in the liver, facilitated by glutathione S-transferase. In this pathway, ACR combines with glutathione, resulting in the generation of N-acetyl-S-cysteine. Subsequently, N-acetyl-S-cysteine undergoes further degradation, resulting in the production of mercapturic ACR acids, which are eventually eliminated through urine. This pathway serves as the main mechanism responsible for the detoxification of ACR.

The main health hazard associated with cigarette smoking arises from the chemicals generated during the combustion of tobacco rather than from nicotine itself [8]. The detrimental consequences of smoking cigarettes stem from a combination of more than seven thousand chemical compounds, among which nicotine is included. According to research conducted by Kenwood et al., cigarette smoking is a prominent factor in the exposure of the United States’ population to acrylamide (ACR) [9]. The combustion of tobacco leads to the formation of ACR, with the smoke emitted by a single cigarette containing an estimated quantity ranging from 0.5 to 4.3 μg of this particular substance [10]. ACR can also be detected in food items that have been baked or fried [11]. As a hydrophilic substance with a small molecular size, ACR undergoes absorption through the digestive tracts of humans as well as animals, subsequently being passively distributed throughout the body. Additionally, ACR can cross the blood–brain barrier, allowing it to directly impact the nervous system and exert its toxic effects [12]. ACR is metabolized in the body through two primary pathways. Under pathway I, concisely, the cytochrome P450 enzyme system, specifically CYP2E1, catalyzes an enzymatic reaction that converts ACR into glycidamide (GA) [13,14]. GA, as a metabolite of ACR, has been shown to induce the formation of DNA adducts, impair neurotransmitter release, provoke degeneration of nerve terminals, and inflict damage to nerve structures. These effects are cumulative and more significant than those of ACR itself. Therefore, it is believed that the main pathway for ACR-induced neurotoxicity is through the actions of GA [13,15]. In pathway II, ACR is biotransformed and catalyzed by liver enzymes, specifically glutathione S-transferase. During this process, ACR combines with glutathione to produce N-acetyl-S-cysteine. Subsequently, it undergoes more degradation, forming mercapturic ACR acids that are eliminated from the body through urine. The second pathway is mostly responsible for the detoxification of ACR (Figure 1) [13].

In 1994, the International Agency for Research on Cancer (IARC) categorized ACR as a Group 2A compound, signifying that it is “probably carcinogenic to humans” [16]. Studies have confirmed that ACR can lead to developmental genotoxicity, neurotoxicity, and potential carcinogenic effects. Specifically, both human and animal studies have demonstrated the neurotoxic effects of ACR [13]. The occurrence of neurological disorders can be attributed to the creation of covalent adducts between ACR and the highly reactive cysteine residues found in the active sites of presynaptic neurons. This interaction deactivates neurons and disrupts the transfer of neurotransmitters, resulting in neurotoxic effects. Additionally, oxidative stress plays a vital role as a biochemical and physiological signaling mechanism, contributing to the neurotoxic effects induced by ACR through both direct and indirect pathways [15,17,18].

**Figure 2:**
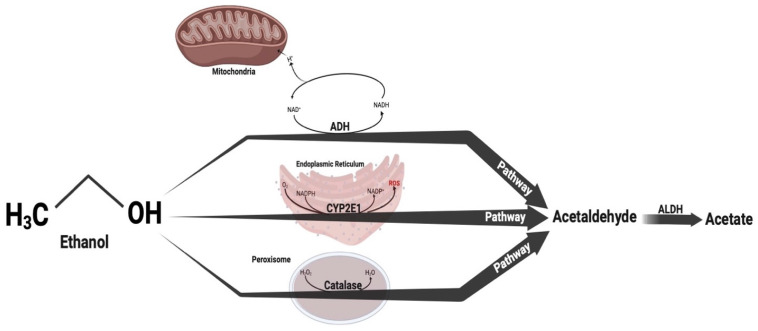
Ethanol is metabolized through three alternative pathways: alcohol dehydrogenase (ADH), microsomal ethanol oxidation system, and catalase. Aldehyde dehydrogenase (ALDH); Nicotinamide Adenine Dinucleotide oxidized (NAD+); Reduced Nicotinamide Adenine Dinucleotide (NADH); NADPH—Reduced Nicotinamide Adenine Dinucleotide phosphate; Nicolinamide Adenine Dinucleotide Phosphate oxidized (NADP+); Reactive Oxygen Species (ROS).

In addition, the “WHO Global Status Report on Alcohol and Health” provided an estimation that around 2.3 billion people are presently consuming alcohol, with an average daily consumption of 32.8 g of pure alcohol [19]. Over the period spanning from 1990 to 2017, the overall per-capita alcohol consumption worldwide experienced an upward trend, and it is projected to further increase by 2030 [20]. Approximately 3 million fatalities were connected to alcohol use in 2016, leading to a total of 132.6 million years of healthy life lost due to disability (DALY) [19]. The effects of alcohol on the CNS differ based on the quantity consumed and the duration of use. Ethanol interacts with numerous cellular targets affected by various neuromodulators within diverse brain neural networks [21]. The detrimental effects of ethanol on the CNS include impaired mnemonic processes, motor function, and neurogenesis inhibition. These effects are partly associated with neuroinflammation, oxidative stress, and excitotoxicity events [22]. Within the brain, alcohol-related damage can be either permanent or reversible. The extent of this damage is influenced by an individual’s lifetime alcohol consumption and associated medical complications [21]. The stimulation of microglia leads to the release of inflammatory mediators, which are triggered by intermittent exposure to ethanol (IEE). Activation also triggers intracellular signaling pathways that stimulate the synthesis of pro-inflammatory cytokines, for instance, tumor necrosis factor-α (TNF-α) and interleukin 1β (IL-1β), in addition to enzymes like inducible nitric oxide synthase (iNOS) and cyclooxygenase-2 (COX-2). These effects are specifically observed in distinct brain regions, such as the cerebellum, parietal association cortex, hippocampus, and entorhinal cortex in rats, leading to adverse consequences on adjacent neurons [23,24,25]. Ethanol undergoes metabolism via three alternative pathways: alcohol dehydrogenase (ADH), microsomal ethanol oxidation system, and catalase. Regardless of the pathway, the primary product of the metabolic reaction is acetaldehyde (Figure 2) [26].

Moreover, both tobacco and alcohol have been identified as health risk behaviors associated with significant, unfavorable health consequences, ranking within the list of the top ten causes of mortality and DALY [27]. The concurrent use of alcohol and tobacco increases the probability of developing specific cancers, as well as cardiovascular and respiratory diseases, to a greater extent than the consumption of either substance alone [28,29]. Tobacco use and excessive alcohol consumption have significant impacts on health, economy, and society. Both substances are addictive and pose health risks. Understanding the connections between these behaviors is crucial, as the combined consumption of tobacco and alcohol amplifies the associated health risks [30]. In vitro studies have shown that the induction of CYP2E1 by ethanol and the depletion of GSH collectively explain the combination effects observed when ethanol and ACR are combined [31]. Conversely, some studies have indicated that consumption of ethanol influences the metabolism of ACR to GA specifically when a high dose of ethanol is consumed [32,33].

In this study, we hypothesized that the co-administration of ACR and intermittent access to alcohol (IAE) consumption could induce neurotoxicity more than ACR or IAE alone by inducing cellular apoptosis, increasing oxidative vulnerability, and promoting brain inflammation and alterations in the histopathological features. To test our hypothesis, we carried out a study to examine the co-administration effect of acrylamide and intermittent alcohol consumption in inducing neurotoxicity. This study involved a comprehensive evaluation of cognitive impairments, susceptibility to the apoptosis process, oxidative stress, neuronal inflammation, and histopathological features in rats.

## 2. Materials and Methods

### 2.1. Animals

The animal study protocol for the current research, conducted at Qassim University, received ethical approval from the Committee of Research Ethics in the Deanship of Scientific Research. The project was assigned the number 23-69-08. Twenty-four adult male Sprague Dawley rats, aged 3 months, and weighing between 150 g and 200 g, were randomly assigned to four equal groups, with each group consisting of 6 rats (*n* = 6). Each polypropylene cage housed three rats. The animal facility at the College of Pharmacy maintained a humidity level of 50% (±5%) and a temperature of 22 °C (±1). The rats were given unrestricted access to food and water, allowing them to consume as desired.

### 2.2. Treatment Groups and Schedule

**Figure 3:**
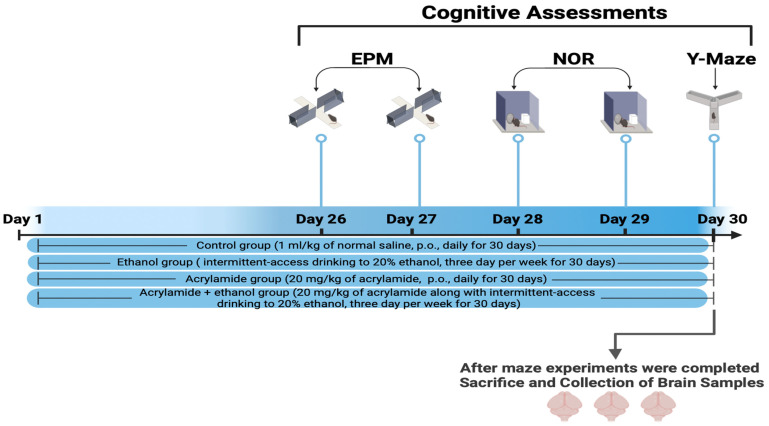
The rats were randomly assigned to four equal groups, with each group consisting of 6 rats: the control group (1 mL/kg of normal saline), the ethanol group (intermittent-access drinking to 20% ethanol), the acrylamide group (20 mg/kg of acrylamide), and the acrylamide + ethanol group (20 mg/kg of acrylamide along with intermittent-access drinking to 20% ethanol). The maze procedures were conducted from days 26 to 30. On day 26, the rats underwent the acquisition phase of the EPM, followed by the retention phase on day 27. On day 28, the rats underwent a habituation phase, and on day 29, they underwent training and test sessions in the NOR maze. Finally, on day 30, the rats underwent a training session in the Y-maze and a test session on the same day as well.

The treatment was administered over a period of one month. The animals were divided into four groups: the control group (1 mL/kg of normal saline), the ethanol group (intermittent-access drinking to 20% ethanol) (ethanol from CHEM-LAB Company, Zedelgem, Belgium), the ACR group (20 mg/kg of acrylamide) (acrylamide from Sigma-Aldrich, St. Louis, MI, USA), and the ACR + ethanol group (20 mg/kg of acrylamide along with intermittent-access drinking of 20% ethanol). The rats had access to 20% ethanol through intermittent drinking sessions lasting 24 h, three times per week on Friday, Sunday, and Tuesday. ACR was orally administered (p.o.) for a period of thirty days after being diluted with normal saline. The experimental dosages and methods of administering ACR at 20 mg/kg orally and ethanol 20 per cent with intermittent-access drinking were determined by referencing previous works [34,35,36]. The maze procedures were conducted during the last five days of the experimental month. The acquisition phase of the EPM started on day 26, and the retention phase took place the following day. On day 28, the rats underwent a habituation phase, and on day 29, they underwent training and test sessions in the NOR maze. On day 30, the rats underwent training sessions in the Y-maze, and on the same day, a test session was also conducted. On the final day of the experiment, after the Y-maze sessions, all the rat brains were collected for analysis using both ELISA and histopathological techniques (Figure 3).

### 2.3. Cognitive Assessments

#### 2.3.1. Elevated Plus Maze (EPM)

**Figure 4:**
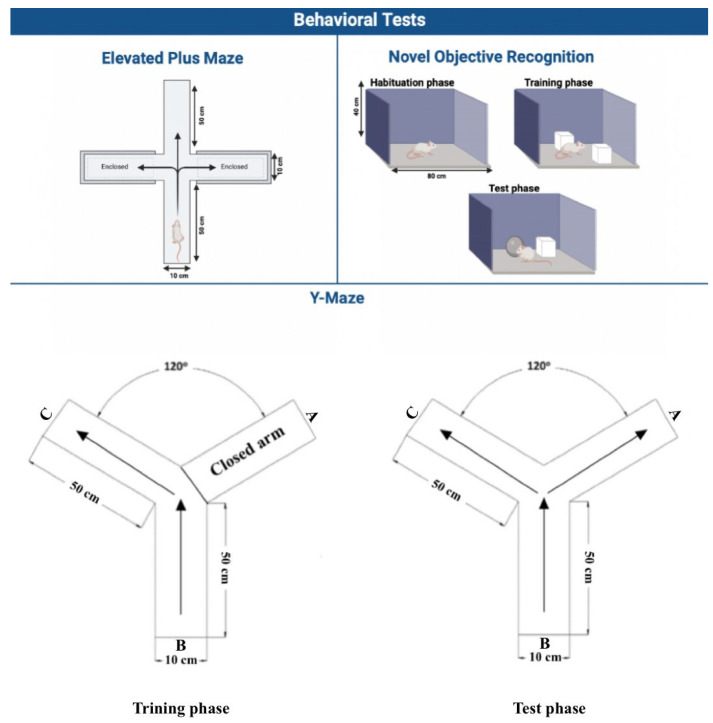
Schematic diagram of EPM, NOR, and Y-maze tests. In the Y-Maze test, arm A represented the novel arm, while the other two arms, B and C, represented the known arms. In the training phase, each rat started from arm B and was allowed to freely explore arms B and C for five minutes, while the novel arm, arm A, remained closed during this phase. In the test phase, each rat started from arm B and was allowed to freely explore all three arms, including the novel arm A, for five minutes.

The EPM test evaluates the ability of animals to learn and remember by measuring their performance in terms of transfer latency (TL) [37]. In summary, the EPM consists of two open arms (50 × 10 cm) and two closed arms (50 × 10 × 40 cm) (Figure 4). It is made of wood and stands fifty centimeters high from the ground. The time it took for a rat to transition from the open arm and enter the closed arm, referred to as TL, was measured as “the time taken by rats to enter the enclosed arm from any one of the open arms” and recorded in seconds [38,39]. The acquisition phase of the EPM started on day 26, and the retention phase took place the following day. On the acquisition phase day, each rat was allowed to explore both the open and closed arms of the maze for two minutes, and the TL was recorded. The exploration started from a designated point at the end of any open arm. On the retention phase day, the same method was repeated, and TL values were recorded as well. The maximum value for TL was set at 90 s. The TL values obtained on day 1 reflected the rats’ performance in acquiring the task, while day 2 assessed their ability to retain the learned behavior.

#### 2.3.2. Novel Object Recognition (NOR)

The NOR test is conducted with a square wooden apparatus with dimensions of 80 cm × 60 cm × 40 cm. It is employed as a means to evaluate the recognition memory of each rat by utilizing two similar cylindrical objects referred to as familiar objects (FO1 and FO2), along with one distinct object (a rectangular object) known as the novel object (NO) [38]. The test procedure consists of three phases over two days. The first phase is the habituation phase, which starts on day 28. The other two phases, the training phase and test phase, occur on the following day (Figure 4). Each phase lasts for five minutes. During the habituation phase, each rat is granted unrestricted exploration within an empty wooden box. On the following day, during the training phase, each rat is given five minutes to explore the wooden box after placement of FO1 and FO2. Each FO is positioned 10 cm away from the wall of the box. After a 4 h interval, the test phase takes place. During this phase, FO1 is replaced with an NO, and each rat is allowed to freely explore the wooden box. The exploration time of familiar (ETFO) and novel (ETNO) objects were recorded during this phase. The exploration time is the duration in which rats direct their nose to an FO or NO within a distance of ≤2 cm, make contact with it, or sniff it. The percentage of discrimination index [PDI = (ETNO − ETFO)/(ETNO + ETFO) × 100] represents the ability of the rats to discriminate between the NO and the FO and was calculated in the test phase [38].

#### 2.3.3. Y-Maze

The Y-maze is a useful tool for assessing an animal’s inclination to explore a new environment and its ability to navigate through a novel arm [38,39]. The Y-maze consists of three arms: A, B, and C. Arm A represented the novel arm, while the other two arms represented the known arms. There was a 120° angle between the arms. Each arm measured 50 cm in length, 30 cm in height, and 10 cm in width. The maze had a Y-shape configuration. The test procedure consisted of two phases on the last day of the experimental timeline. The first phase was the training phase, followed by a test phase after a 4 h interval (Figure 4). In the training phase, each rat started from arm B and was allowed to freely explore arms B and C for five minutes to become familiar with them, while the novel arm, arm A, remained closed during this phase. Following a four-hour interval, the test session commenced, and each rat was granted unrestricted access to explore all three arms for a duration of five minutes. During this phase, the number of entries into two known arms (NEKA) as well as a novel arm (NENA) and the time spent in each arm were documented. Lastly, the percentage of the duration of time spent in the novel arm (TSNA) was calculated [TSNA = (time spent in the novel arm/total time spent in all arms) × 100] using methodologies outlined in previous research studies [38,39].

### 2.4. Biochemical Analysis—Enzyme-Linked Immunosorbent (ELISA) Assay

#### 2.4.1. Brain Isolation

After completing the maze experiments on day 30, a humane euthanasia (ketamine—100 mg/kg and xylazine—10 mg/kg) method involving cervical dislocation was performed on each rat. Following euthanasia, the brains of all rats were carefully collected and then cut identically into two sagittal halves. The first halves were homogenized using ice-cold phosphate-buffered saline at a temperature of 4 °C. Subsequently, the homogenate was centrifuged at a speed of 4000 revolutions per minute for a duration of 10 min, leading to the separation of a portion (aliquot). This aliquot was stored at −80 °C. The biuret colorimetric method was employed to determine the total protein content in each sample of brain tissue homogenate. The second halves of the brain were placed in labeled containers filled with 10% buffered formalin for fixation.

#### 2.4.2. Neuronal Inflammation

In order to evaluate the neuroinflammatory effect of IAE and ACR on the CNS, this study focused on analyzing the levels of three specific inflammatory parameters in brain homogenates. To assess inflammatory parameters, we employed rat double antibody sandwich ELISA kits to measure PGE2 (MBS 7606497), TNF-α (MBS 2507393), and NF-κB (MBS 453975). The assays were performed according to the assay kit instructions provided by MyBioSources (San Diego, CA, USA).

#### 2.4.3. Neuronal Apoptosis

Furthermore, we evaluated the concentrations of Bcl2, which is an anti-apoptotic protein, and Caspase-3 and Bax, which are pro-apoptotic proteins from the brain tissue homogenate. Rat double antibody sandwich ELISA kits for Bcl2 (MBS 452319), Bax (MBS 2703209), and Caspase-3 (MBS 729893) were utilized to analyze apoptosis parameters. The ELISA kits used in this study were acquired from MyBioSources (San Diego, CA, USA), and we followed the instructions provided with the kits.

#### 2.4.4. Neuronal Oxidative Stress

In our current study, we assessed oxidative stress by measuring specific oxidative parameters, such as MDA as an oxidative marker, and GSH and catalase as antioxidant markers, in brain homogenates. To accomplish this, we employed rat double antibody sandwich ELISA kits for MDA (MBS 738685), GSH (MBS 2540412), and CAT (MBS 2704433). The assays were performed according to the assay kit instructions provided by MyBioSources (San Diego, CA, USA).

#### 2.4.5. Histopathological Analysis

For tissue processing, we used the Sakura Histo-Tek VP1 tissue processor and followed a protocol in sequential multiple sessions, which includes formalin, distilled water, ethanol (75–100%), xylene, and paraffin. The paraffin blocks were cut by using HistoCore AUTOCUT (Leica Biosystems, IL, USA) to a thickness of 4.5 microns in order to form thin sections. Finally, the slides were stained and coverslipped with the help of the Tissue-Tek Prisma Plus and Tissue-Tek Film (Sakura Finetek, Torrance, CA, USA) automated machine.

### 2.5. Statistical Analysis

The data were summarized by calculating the average and variability of the results. GraphPad version 9.5.0 (GraphPad Software Inc., San Diego, CA, USA) was utilized to carry out a one-way ANOVA analysis, which determined the significance levels among the different groups. Subsequently, a Tukey–Kramer post hoc test was conducted to evaluate the significance levels between specific pairs of groups. Additionally, to compare the FO1 and NO groups in the NOR test, a Student’s unpaired *t*-test was utilized. A *p*-value of ≤0.05 was deemed as statistically significant.

## 3. Results

### 3.1. Effect of ACR and IAE on Transfer Latency (TL) Time of Elevated Plus-Maze (EPM) Test

**Figure 5:**
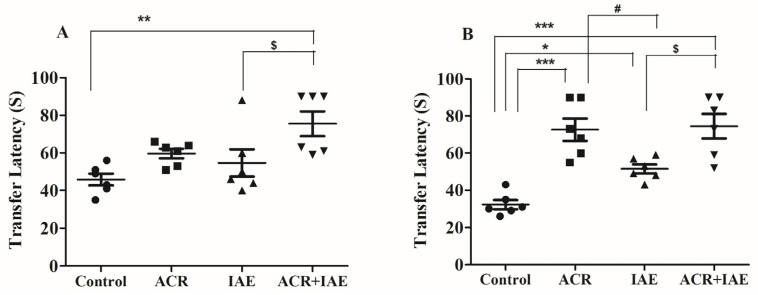
Investigation of the impact of ACR, IAE, and ACR + IAE on rats utilizing the EPM. Chart (**A**) displays the TL of rats on the first day, while chart (**B**) represents the TL on the second day. The findings are displayed as the mean ± SEM (*n* = 6). A one-way ANOVA was performed for the first day TL [F(3,20) = 5.60, *p* < 0.01] and second day TL [F(3,20) = 17.25, *p* < 0.001]. The Tukey–Kramer multiple comparisons test was utilized for post hoc analysis. Significance levels were indicated as * *p* < 0.05, ** *p* < 0.01, and *** *p* < 0.001 compared to the control group. Additionally, # *p* < 0.05 was significant compared to the ACR group, and $ *p* < 0.05 was significant compared to the IAE group.

The TL time (in seconds) was measured for all rats who underwent treatment utilizing the EPM test. A shorter TL value in the test indicated an enhancement in their learning and spatial memory capabilities. One-way ANOVA tests revealed significant differences on both the 1st day [F(3,20) = 5.61, *p* < 0.01] and the 2nd day [F(3,20) = 17.25, *p* < 0.001], as shown in Figure 5. Post hoc Tukey–Kramer multiple comparisons tests revealed that on the 1st day, the ACR + IAE group showed a significantly long TL time (75.50 ± 6.51, *p* < 0.01) relative to the control group (45.83 ± 3.10). Similarly, the ACR + IAE group had a markedly prolonged TL time (*p* < 0.05) compared to the IAE group (54.67 ± 7.22). The extended TL time illustrated the learning difficulties in rats treated with ACR + IAE. No other noteworthy changes were found among the remaining treated groups on the 1st day (Figure 5A). On the 2nd day, there were markedly prolonged TL times in the ACR group (72.67 ± 6.04, *p* < 0.001), IAE group (51.50 ± 2.45, *p* < 0.05), and ACR + IAE group (74.50 ± 6.59, *p* < 0.001), when compared to the control (32.33 ± 2.45), highlighting the memory deficits in these groups. Additionally, the ACR group showed a significantly prolonged TL time (*p* < 0.05) in comparison to the IAE group, and the group treated with both ACR and IAE exhibited a significantly prolonged TL time (*p* < 0.05) in relation to the IAE group. On the second day, there were no notable distinctions between the ACR group and the ACR + IAE group (Figure 5B). These changes indicated that IAE rats exhibited minimal alterations in memory.

### 3.2. Effect of ACR and IAE on Targeted Cognitive Parameters in the Novel Object Recognition (NOR) Test

**Figure 6:**
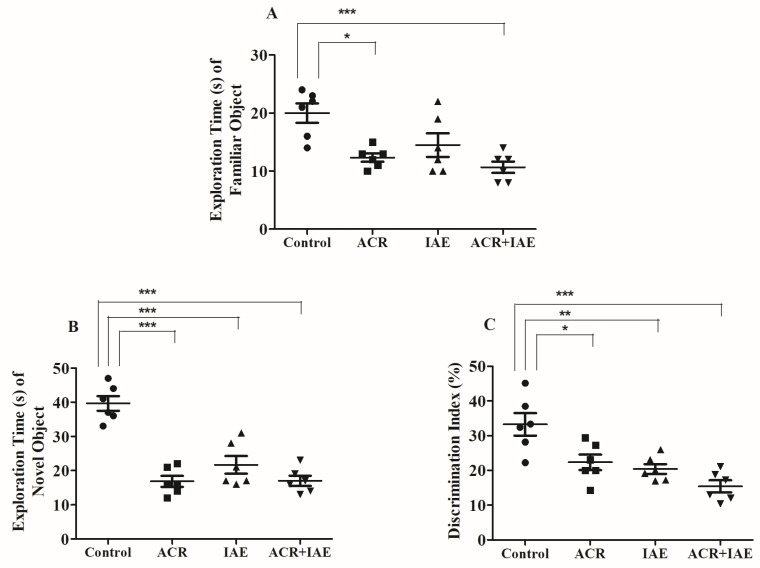
The impact of ACR, IAE, and ACR + IAE on rats was examined utilizing the NOR test. Chart (**A**) represents the ETFO, chart (**B**) displays the ETNO, and chart (**C**) indicates the PDI of rats in the NOR test. The findings are presented as the mean ± SEM (*n* = 6). The data were analyzed using one-way ANOVA, yielding significant findings for ETFO [F(3,20) = 7.97, *p* < 0.01], ETNO [F(3,20) = 18.92, *p* < 0.001], and PDI [F(3,20) = 11.04, *p* < 0.001]. The Tukey–Kramer multiple comparisons test was utilized for post hoc analysis. The levels of significance were indicated as * *p* < 0.05, ** *p* < 0.01, and *** *p* < 0.001 in comparison to the control group.

During the test session, notable variations among all groups were detected through the analysis of a one-way ANOVA [F(3,20) = 7.97, *p* < 0.01] in ETFO (Figure 6A). Further comparisons using the Tukey–Kramer test unveiled a noteworthy disparity in the exploration times for a familiar object (ETFO) between the control group (20.00 ± 1.65) and the ACR group (12.33 ± 0.71, *p* < 0.05). Likewise, a remarkable difference in ETFO was noted between the control group and the ACR + IAE group (10.67 ± 0.99, *p* < 0.001). Both treatments resulted in a deficit in the animals’ exploration capability. There were no distinctions observed among the other groups.

Figure 6B shows significant variations in exploration times for novel object (ETNO) among all groups [F(3,20) = 18.92, *p* < 0.001]. Additionally, in relation to the control group (39.67 ± 2.16), there was a remarkable difference (*p* < 0.001) in ETNO with the ACR group (16.83 ± 1.60), IAE group (21.67 ± 2.60), and ACR + IAE group (17.00 ± 1.48). The remaining groups did not exhibit any noteworthy differences. Similarly, the exploration capability of a new object was affected by all treatments, but no significant differences were observed between any of these administrations.

Concerning the PDI, its measurement using one-way ANOVA illustrated a remarkable reduction [F(3,20) = 11.04, *p* < 0.001] (Figure 6C). A notable distinction was observed in the PDI value match between the control group (33.27 ± 3.25) and other treated groups, such as the ACR group (22.34 ± 2.24, *p* < 0.05), IAE group (20.40 ± 1.43, *p* < 0.01), and ACR + IAE group (15.40 ± 1.73, *p* < 0.001). The remaining groups did not exhibit any noteworthy differences. These results highlighted that the recognition ability of rats was impaired in all groups, but no significant differences were noted between any of the treatments.

### 3.3. Effect of ACR and IAE on Targeted Cognitive Parameters in Y-Maze Test

The effects of ACR, IAE, and ACR + IAE on rat behaviors, specifically their exploration of a new environment and their ability to navigate through a novel arm, were assessed through the Y-maze test. The analysis during the test session primarily studied the number of entries into both the novel and known arms (NENA and NEKA, respectively), along with the duration of time spent in the novel arm (TSNA).

**Figure 7:**
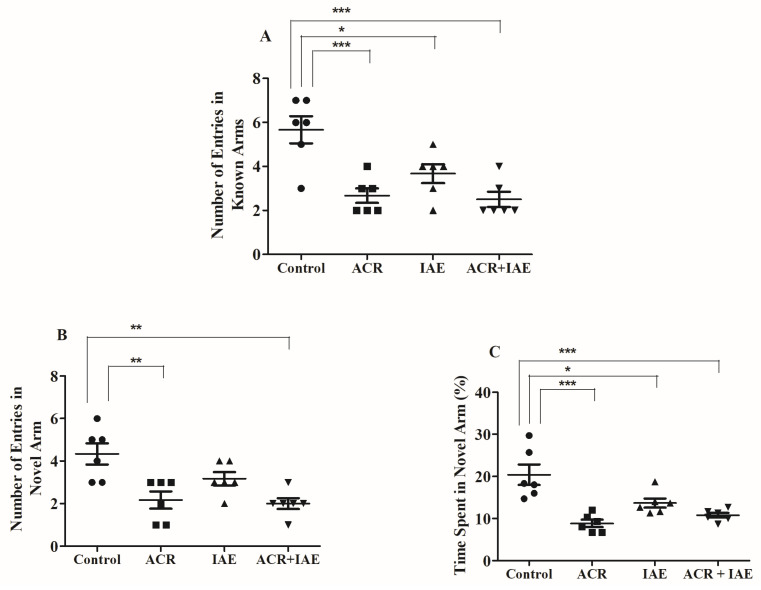
The effects of ACR, IAE, and ACR + IAE on rats were investigated using the Y-maze test. Chart (**A**) represents the NEKA, chart (**B**) represents the NENA, and chart (**C**) shows the percentage of TSNA. The results are presented as mean ± SEM (*n* = 6). One-way ANOVA tests were conducted to analyze the data, with significant results obtained for NEKA [F(3,20) = 10.82, *p* < 0.001], NENA [F(3,20) = 8.17, *p* < 0.001], and the TSNA [F(3,20) = 12.57, *p* < 0.001]. The Tukey–Kramer multiple comparison test was utilized for post hoc analysis. Significance levels were indicated as * *p* < 0.05, ** *p* < 0.01, and *** *p* < 0.001 in comparison to the control group.

Figure 7 showcases the analysis performed to assess the impacts of ACR, IAE, and ACR + IAE on the NEKA, NENA, and TSNA. A one-way ANOVA was conducted, revealing significant results on the NEKA (F(3,20) = 10.82, *p* < 0.001), NENA (F(3,20) = 8.17, *p* < 0.001), and the percentage of TSNA (F(3,20) = 12.57, *p* < 0.001). In Figure 7A, ACR administration significantly decreased NEKA (2.67 ± 0.33, *p* < 0.001) relative to the normal group (5.67 ± 0.61). Similarly, IAE administration resulted in a reduction in NEKA (3.67 ± 0.42, *p* < 0.05) associated with the normal rats. The combined administration of ACR + IAE also led to a significant decrease in NEKA (2.50 ± 0.34, *p* < 0.001) compared to the control. However, no significant differences were observed in NEKA when comparing the individual treatments of ACR or IAE to the combined treatment of ACR + IAE.

Regarding Figure 7B, which depicts the NENA, noteworthy distinctions were observed in the ACR group (2.17 ± 0.40, *p* < 0.01) and the ACR + IAE group (2.00 ± 0.26, *p* < 0.01) in relation to the control group (4.33 ± 0.49). However, there were no remarkable variations detected in NENA between the control group and the IAE group (3.17 ± 0.31). Furthermore, no significant differences in NENA were observed between the ACR + IAE group and the IAE group, or between the ACR group and the ACR + IAE group. Similarly, there were no remarkable variations found between the ACR group and the IAE group.

Finally, in Figure 7C, ACR administration significantly decreased the TSNA (8.83 ± 0.87, *p* < 0.001) likened to the control rats (20.39 ± 2.42). Similarly, IAE administration led to a reduction in TSNA (13.67 ± 1.09, *p* < 0.05) when compared to the normal group. The combined administration of ACR + IAE led to a significant reduction in TSNA (10.78 ± 0.58, *p* < 0.001) compared to the control group. However, no significant differences were found in the TSNA when comparing the individual treatments of ACR or IAE to the combined treatment of ACR + IAE.

All the preceding findings emphasize the presence of spatial working memory deficits across all treatments, with the degree of deficits being comparable among them.

### 3.4. Effect of ACR and IAE on Neuro-Inflammatory Mediators in the Rat Brain

**Figure 8:**
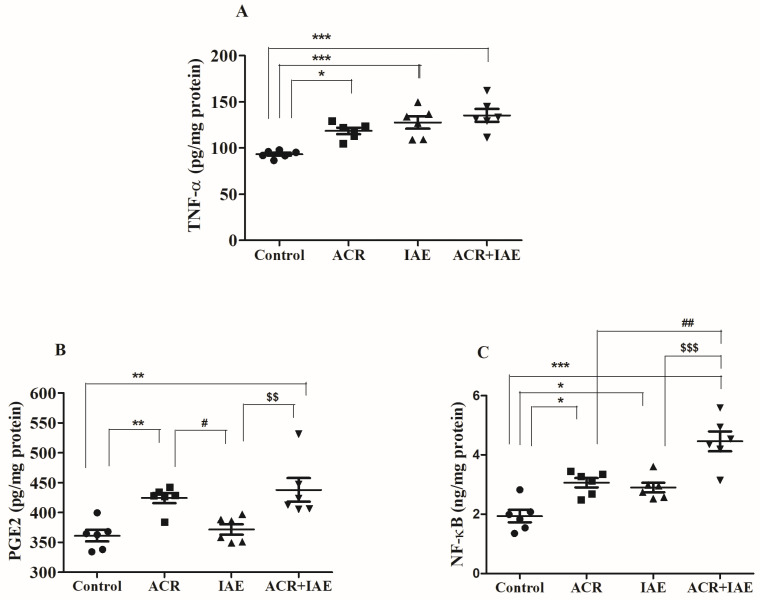
The effects of ACR, IAE, and ACR + IAE on inflammatory factors in rats, including (**A**) NF-κB, (**B**) PGE2, and (**C**) TNF-α, were examined. The results presented as mean ± SEM (*n* = 6) were subjected to one-way ANOVA tests for analysis. Notable variations were identified for TNF-α (F(3,20) = 12.63, *p* < 0.001), PGE2 (F(3,20) = 9.16, *p* < 0.001), and NF-κB (F(3,20) = 20.85, *p* < 0.001). The Tukey–Kramer multiple comparisons test was utilized for post hoc analysis. The levels of significance were indicated as * *p* < 0.05, ** *p* < 0.01, and *** *p* < 0.001 compared to the control group; # *p* < 0.05 and ## *p* < 0.01 compared to the ACR group; and $$ *p* < 0.01 and $$$ *p* < 0.001 compared to the IAE group.

Targeted neural-inflammatory markers, such as prostaglandin E2 (PGE2), nuclear factor kappa B (NF-κB), and TNF-α, were examined to evaluate the influence of IAE on ACR-induced neuronal inflammatory insults in brain tissues (Figure 8).

Figure 8A revealed notable variations in TNF-α levels (pg/mg protein) [F(3,20) = 12.63, *p* < 0.001] across the groups subjected to treatment. The ACR, IAE, and ACR + IAE groups exhibited numerically substantial elevations in comparison to the control group (93.26 ± 1.67). These increases in TNF-α levels were correlated with inflammatory triggers in brain tissues. More precisely, the ACR group exhibited a noteworthy increase (118.50 ± 3.54, *p* < 0.05) as opposed to the control group. Similarly, the IAE group exhibited a significant increase (127.70 ± 6.59, *p* < 0.001) in relation to the control group. As anticipated, the ACR + IAE group displayed a significant increase (135.50 ± 6.92, *p* < 0.001) compared to the control group. There were no significant changes in TNF-α levels observed when comparing the treatment with ACR alone or IAE alone to the combined treatment (ACR + IAE).

Furthermore, after conducting one-way ANOVA tests, significant alterations in PGE2 levels (pg/mg protein) were observed [F(3,20) = 9.16, *p* < 0.001] among the experimental groups (Figure 8B). Subsequently, the Tukey–Kramer multiple comparisons test was conducted. As anticipated, the ACR + IAE group exhibited a significant increase (437.8 ± 19.75, *p* < 0.01) when compared to the control group (361.2 ± 9.66). Additionally, it exhibited a higher level (*p* < 0.01) in comparison to the IAE group (371.7 ± 8.67). Furthermore, the ACR group (424.4 ± 8.41, *p* < 0.01) demonstrated a noticeable increase in PGE2 levels in comparison to the control group and also a higher level (*p* < 0.05) than the IAE group. Inflammatory alterations with PGE2 levels were found in both the ACR and ACR + IAE treatments, but not in the IAE treatment.

A significant rise in the NF-κB biomarker level (ng/mg protein) was observed [F(3,20) = 20.85, *p* < 0.001], as shown in Figure 8C. The ACR, IAE, and ACR + IAE groups demonstrated notable increases relative to the control group (1.94 ± 0.21). Specifically, the ACR group demonstrated a remarkable elevation (3.06 ± 0.16, *p* < 0.05) compared to the control group. Likewise, the IAE group exhibited a notable and statistically significant elevation (2.90 ± 0.16, *p* < 0.05) in comparison to the control group. As expected, the ACR + IAE group demonstrated a substantial and statistically significant increase (4.46 ± 0.33, *p* < 0.001) when contrasted with the control group. Moreover, the ACR + IAE group displayed a significant increase (*p* < 0.01) compared to the ACR group, as well as a significantly higher level (*p* < 0.001) compared to the IAE group. These significant changes clearly support the elevation of inflammatory responses in the combined administration compared to either ACR or IAE alone.

### 3.5. Effect of ACR and IAE on Apoptosis Parameters in the Rat Brain

**Figure 9:**
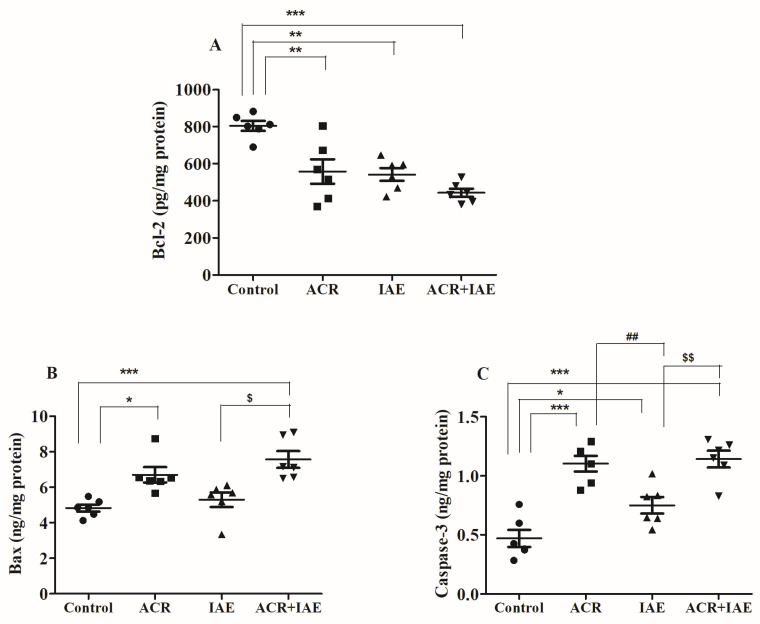
Effects of ACR, IAE, and ACR + IAE on various factors related to apoptosis, including (**A**) Bcl-2, (**B**) Bax, and (**C**) Caspase-3 in rats. The findings, presented as the mean ± SEM (*n* = 6), were analyzed using one-way ANOVA tests. Significant differences were observed for Bcl-2 (F(3,20) = 13.83, *p* < 0.001), Bax (F(3,20) = 10.34, *p* < 0.001), and Caspase-3 (F(3,20) = 20.79, *p* < 0.001). The Tukey–Kramer multiple comparisons test was utilized for post hoc analysis. The levels of significance were indicated as * *p* < 0.05, ** *p* < 0.01, and *** *p* < 0.001 compared to the control group. Additionally, ## *p* < 0.01 compared to the ACR group, and $ *p* < 0.05 and $$ *p* < 0.01 compared to the IAE group.

The study consisted of multiple phases, including an analysis of specific apoptosis biomarkers such as B-cell lymphoma 2 (Bcl-2), Bcl-2 associated X protein (Bax), and cysteinyl aspartate specific proteinase 3 (Caspase-3). The objective of this analysis was to assess the effects of IAE on ACR-induced apoptosis in brain tissues, as illustrated in Figure 9.

Notable changes [F(3,20) = 13.83, *p* < 0.001] were noted in the levels of the anti-apoptotic protein Bcl-2 (pg/mg protein) among all groups that received treatment (Figure 9A). The ACR, IAE, and ACR + IAE groups exhibited meaningful modifications when compared to the control group (804.5 ± 26.84). Specifically, the ACR group demonstrated a significant decline (558.4 ± 66.34, *p* < 0.01) in comparison to the control group. Similarly, both the IAE group (542.3 ± 34.74, *p* < 0.01) and the ACR + IAE group (443.4 ± 22.22, *p* < 0.001) displayed a decrease. The lowering of Bcl-2 levels indicates a reduction in anti-apoptotic capability due to the administration of ACR and IAE. Furthermore, the higher significance level (*p* < 0.001) observed with ACR + IAE demonstrates a greater effect. No significant changes were observed in Bcl-2 levels when comparing treatment with ACR alone or IAE alone to the combined treatment (ACR + IAE).

Significant variations [F(3,20) = 10.34, *p* < 0.001] were detected in the levels of the pro-apoptotic protein Bax (ng/mg protein) among all treated groups (Figure 9B). Compared to the control group (4.83 ± 0.20), both the ACR-treated group (6.70 ± 0.43, *p* < 0.05) and the ACR + IAE-treated group (7.57 ± 0.47, *p* < 0.001) displayed noticeable and significant increases. The higher significance level observed with ACR + IAE indicates an additional influence of IAE in the combined administration. As expected, the ACR + IAE group demonstrated a noteworthy increase when compared to the IAE-alone group (5.30 ± 0.41, *p* < 0.05). There were no remarkable changes detected in Bax levels between the control group and the IAE group, or between the ACR and ACR + IAE groups.

The levels of the pro-apoptotic protein Caspase-3 (ng/mg protein) showed statistically remarkable effects [F(3,20) = 20.79, *p* < 0.001] across all groups (Figure 9C). The ACR, IAE, and ACR + IAE groups exhibited significant changes in relation to the control group (0.47 ± 0.07). The ACR group displayed a remarkable rise (1.14 ± 0.07, *p* < 0.001) relative to the control group as well. Similarly, the IAE group illustrated a substantial elevation (0.75 ± 0.07, *p* < 0.05) compared to the control group. Additionally, the ACR + IAE group demonstrated a significant increase (1.14 ± 0.07, *p* < 0.001) compared to the control group. The elevation in Caspase-3 levels across all treated groups suggests an increase in apoptotic activity. Furthermore, the ACR group showed a significant rise (*p* < 0.01) in comparison to the IAE group. Consistent with expectations, the ACR + IAE group also demonstrated a significant increase (*p* < 0.01) when compared to the IAE group. This highlights that there was a lower influence of IAE on changes in Caspase-3 levels in combined administration. In contrast to the earlier findings, no significant changes were observed in Caspase-3 levels between the ACR group and the ACR + IAE group.

### 3.6. Effect of ACR and IAE on Oxidative Parameters in the Rat Brain

**Figure 10:**
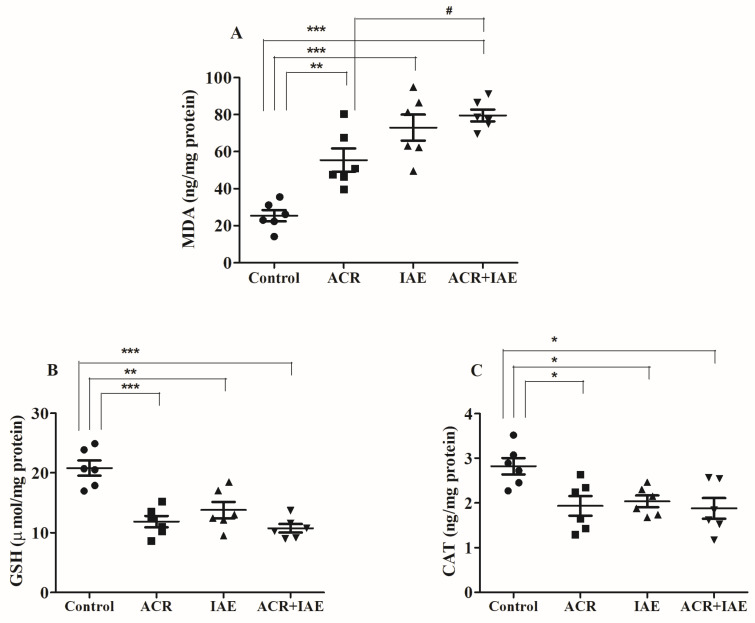
Analysis of ACR, IAE, and ACR + IAE on oxidative factors, specifically (**A**) MDA, (**B**) GSH, and (**C**) CAT, in rats. The findings, presented as the mean ± SEM (*n* = 6), were analyzed using one-way ANOVA tests. Significant differences were observed for MDA (F(3,20) = 21.62, *p* < 0.001), GSH (F(3,20) = 16.48, *p* < 0.001), and CAT (F(3,20) = 5.07, *p* < 0.01). The Tukey–Kramer multiple comparisons test was utilized for post hoc analysis. The levels of significance were indicated as * *p* < 0.05, ** *p* < 0.01, and *** *p* < 0.001 compared to the control group and # *p* < 0.05 compared to the ACR group.

The study analyzed specific oxidative biomarkers, such as malondialdehyde (MDA), and antioxidant biomarkers, such as reduced glutathione (GSH) and catalase (CAT), to assess the influence of IAE, ACR, and ACR + IAE on induced oxidative stress in the neural tissue of the brain (Figure 10).

After conducting one-way ANOVA tests, notable disparities were found in the levels of MDA (ng/mg protein) across all treated groups [F(3,20) = 21.62, *p* < 0.001] (Figure 10A). Notably, ACR, IAE, and ACR + IAE groups exhibited significant alterations related to the control (25.35 ± 3.05), indicating the development of oxidative stress in the rat’s brain. Specifically, the ACR group exhibited a notable rise (55.41 ± 6.27, *p* < 0.01) in comparison with the control group. Similarly, both the IAE group (72.89 ± 7.02, *p* < 0.001) and the ACR + IAE group (79.53 ± 3.22, *p* < 0.001) showed significant elevations in MDA levels. As anticipated, there was a noticeable and substantial increase (*p* < 0.05) in MDA levels in the ACR + IAE group in comparison to the group treated with ACR. This clearly evidences the influence of IAE in the combined administration. No noticeable alterations in MDA levels were observed among the ACR + IAE group and the IAE group.

In Figure 10B, noticeable variances were observed in the levels of GSH (μmol/mg protein) among all treated groups [F(3,20) = 16.48, *p* < 0.001]. The ACR, IAE, and ACR + IAE groups all exhibited notable decreases in comparison to the control group (20.81 ± 1.28). Additionally, the ACR group showed a significant demotion (11.85 ± 0.97, *p* < 0.001) in comparison to the control group. Likewise, the IAE group (13.78 ± 1.37, *p* < 0.01) and the ACR + IAE group (10.75 ± 0.71, *p* < 0.001) both demonstrated reductions in comparison to the control group. The significant reduction in GSH levels indicates that both ACR and IAE challenge the antioxidant system in brain tissues. Nevertheless, no remarkable alterations were observed in GSH levels when comparing the treatment with ACR alone or IAE alone to the combined treatment (ACR + IAE).

Furthermore, significant changes in CAT levels (ng/mg protein) were observed [F(3,20) = 5.07, *p* < 0.01] (Figure 10C). All treatment groups (ACR, IAE, and ACR + IAE) exhibited statistically significant reductions (*p* < 0.05) in CAT levels in comparison to the control group (2.82 ± 0.18). Specifically, CAT levels in the ACR group were measured as 1.93 ± 0.22, in the IAE group as 2.03 ± 0.13, and in the ACR + IAE group as 1.88 ± 0.23. In parallel to the GSH results, the reduction in CAT levels across all treatments further supports the decline in the brain’s antioxidant system. There were no notable alterations observed in CAT levels when comparing all the treated groups (ACR, IAE, and ACR + IAE).

### 3.7. Effect of ACR and IAE on the Histopathology of the Rat Brain

**Figure 11:**
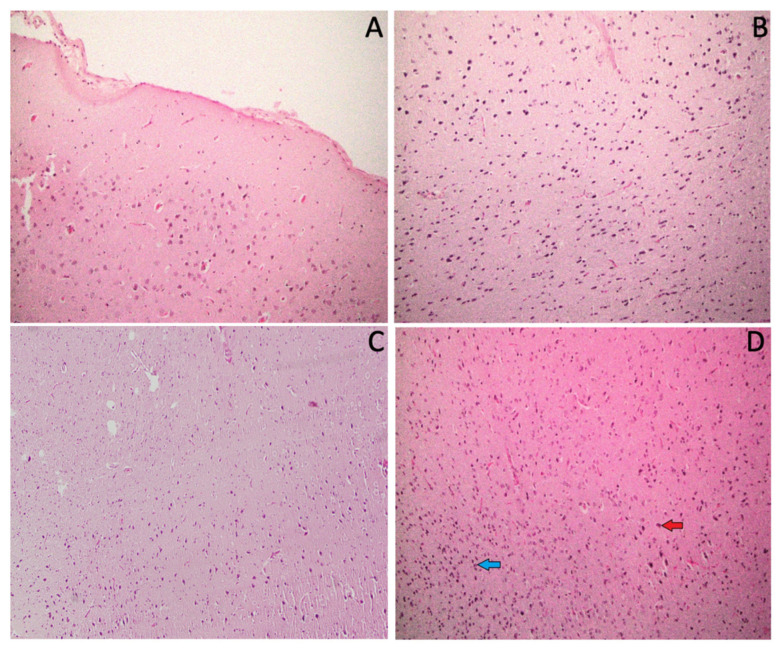
Photomicrograph of a histopathology section using hematoxylin and eosin. (**A**) Normal group: the section demonstrates neurons and glial cells with unremarkable morphological changes. (**B**) ACR group: the section shows reactive gliosis that presents as a reaction to the brain injury. (**C**) IAE group: section shows cellular cortex layer. This cellularity is formed by many glial cells (gliosis) with scattered red neurons in between. (**D**) ACR + IAE group: the section shows red neurons (red arrow) with marked reactive gliosis, mainly astrocytes (blue arrow). These pictures are taken from the cerebral cortex (H&E [×200 magnification]).

The histopathological features of the normal control group show normal brain histological features, and the photomicrograph (Figure 11A) showed neurons and glial cells with unremarkable morphological changes. In contrast, the histopathological features of the ACR group (Figure 11B) show reactive gliosis (increased number of reactive glial cells, mainly astrocytes). These changes indicate that there is an injury that occurs to the neurons. Furthermore, the histopathological features of the IAE group show the mild form of red neurons and gliosis as shown in Figure 11C. In comparison to the ACR group and ACR + IAE group, these changes are very mild; however, evidence of neuronal injury is still there.

Moreover, the histopathological features of the ACR + IAE group show features similar to the ACR group but more aggressive since the red neurons as well as the reactive gliosis become more prominent than the ACR group, as shown in Figure 11D. This indicates that this group has more brain injury than in the ACR group.

## 4. Discussion

ACR is capable of traversing the blood–brain barrier and exerting a direct influence on the nervous system by creating covalent adducts with cysteine residues in presynaptic neurons. This process inhibits the functioning of neurons and disrupts the transmission of neurotransmitters, resulting in neurotoxic effects. Oxidative stress also contributes to ACR-induced neurotoxicity [12,15,17,18]. Ethanol interacts with various cellular targets in the brain and has detrimental effects on cognitive function, motor function, and neurogenesis inhibition. Neuroinflammation, oxidative stress, and excitotoxicity are involved in ethanol’s harmful effects. Intermittent ethanol exposure activates microglia, leading to the release of inflammatory mediators and the synthesis of pro-inflammatory cytokines and enzymes. These effects are observed in specific brain regions, such as cerebellum, hippocampus, entorhinal cortex, striatum, and neocortex, impacting adjacent neurons [21,22,23,24,25]. In the current study, we examined the impact of simultaneously administering ACR and IAE on the induction of neurotoxicity. The study encompassed a comprehensive evaluation of cognitive impairments, responsiveness to apoptosis, oxidative stress, histopathological features, and neuronal inflammation in the brain tissues of rats.

The effects of ACR and alcohol on behavior were assessed using specific tasks including the EPM, NOR, and Y-Maze. The drug administration was conducted throughout thirty days and maze procedures were performed from days 26 to 30. The animals were categorized into four groups: the control group, the ethanol group, the ACR group, and the ACR + ethanol group. The study revealed that ACR exhibited neurotoxic properties and adversely affected learning and memory abilities [40]. In a prior investigation, it was noticed that mice exhibited memory impairment following four weeks of intermittent access to 20% alcohol [41]. Similarly, in the present study, it was also noticed that rats exhibited memory impairment following intermittent access to 20% alcohol, as assessed using tests including EPM, NOR, and Y-maze.

Firstly, the EPM test was conducted over a period of two days, beginning on day 26 of the treatment timeline, with the subsequent day designated as the retention phase. The EPM test is a widely used neutral behavioral model to study the ability of animals to learn and remember [38,42]. Normally, animals tend to avoid open and elevated areas while exhibiting a preference for closed and dark areas. Additionally, shorter TL values indicate improved spatial memory [42]. The study findings revealed that on the first day of the EPM test, the ACR + IAE co-administration group exhibited significantly longer TL values and a decline in spatial memory in relation to the control group. On the second day, all treated groups had larger TL values than the control group. Similar to the first day, the second day’s results also indicated a deterioration in spatial memory. The IAE-alone group had smaller TL values than both the ACR-alone group and the ACR + IAE group. However, there were no notable distinctions observed between the ACR group and the ACR + IAE group. Therefore, the EPM test results indicate significant impairment in the behavior and cognitive abilities of the co-administration ACR + IAE group and the ACR-alone group.

To assess the recognition memory of rats, we employed the NOR test. This test comprised three phases conducted on days 28 and 29 of the treatment timeline: habituation, training, and testing. Each rat spends five minutes in each phase. The duration of exploration for both the familiar (ETFO) and novel objects (ETNO) was documented, and the discrimination ability was evaluated using the PDI [38]. During the testing phase, rats that allocated more time to exploring the FO exhibited memory retention for the NO presented in the initial trial. Conversely, if the rats spent an equal length of time dedicated to examining both objects, it indicated a lack of recollection or memory for the FO observed during the initial trial [43,44]. Our findings demonstrate a notable reduction in the ETFO in both the ACR group and the ACR + IAE group relative to the control group during the test session. Likewise, all treatment groups exhibited a noteworthy reduction in the ETNO when compared to the control group. Furthermore, a substantial decline was noted in the PDI value among all treatment groups when compared to the control group. Overall, our results provide evidence of impairments in working memory and discrimination ability in all treatment groups. These outcomes align with our previous research, indicating that animals tend to approach and spend more time exploring the unfamiliar novel object (NO) when given a choice between the NO and the FO [45,46]. In our previous study, we observed that normal animals typically approach and spend more time exploring the NO when given a choice between it and an FO, regardless of the treatment. Based on this behavior, we can infer the abnormal outcomes in treated rats by comparing their behavior to that of the normal group [45,46]. In an earlier report, adolescent male and female rats were subjected to multiple weekly sessions where they were continuously exposed to drinking water containing 20% ethanol. When tested using the NOR test, these rats showed impaired recognition of an NO compared to the control group. This impairment was reflected in reduced discrimination values during the test phase, while no notable differences were observed in exploration times during the training phase [47]. Our current study followed an intermittent-access schedule to 20% alcohol, with exposure occurring three days per week [36]. Additionally, our study extended with further information regarding the effects of ACR and the combined effects of ACR + IAE.

Through the Y-maze test, the impact of co-administration of ACR and IAE on various cognitive parameters was examined. The parameters assessed in this study encompassed the number of entries made in both the known (NEKA) and novel arms (NENO) of the maze, as well as the proportion of time dedicated to exploring the novel arm. All treatment groups demonstrated a decline in NEKA. Furthermore, both the ACR and ACR + IAE groups exhibited a decrease in NENA. Additionally, rats in all treatment groups spent less time in the novel arm, suggesting impairments in working memory across all treatment groups. These results align with a previous study that investigated the impact of ACR on memory impairment using the Y-maze platform. The research revealed a notable decrease in the fraction of accurate arm alternation due to ACR, which suggests impaired spatial memory [48]. These results align with a prior investigation, as intermittent alcohol administration was found to have a negative effect on spatial memory. This was evident through a decrease in the percentage of correct alternations in the Y-maze. Additionally, spatial memory impairment was observed in both the 25% and 5% ethanol solution groups, where alcohol exposure occurred once every 3 days, when compared to the control group [49].

Regarding neuroinflammatory mediators, NF-κB plays a regulatory role in various target genes associated with cell apoptosis and proliferation. The NF-κB signaling pathway is open to cross-talk and impacts multiple signaling pathways. ACR can induce apoptosis by activating the NF-κB signaling pathway driven by Mitogen-Activated Protein Kinase (MAPK), leading to cytotoxic effects [50,51]. The activation of NF-κB initiates inflammation and immune responses by upregulating the expression of genes encoding proinflammatory cytokines such as TNF-α, IL-1β, IL-6, and COX-2 [52,53,54]. Previous studies have reported that administering ACR to experimental animals at different doses, such as 20 mg/kg, induces a substantial rise in the levels of inflammatory cytokines, such as TNF-α, IL-1β, and IL-6 [55]. Moreover, PGE2 plays a crucial role in the process of inflammation by directly inducing vasodilation and activating various other inflammation mediators [56]. On the other hand, intermittent exposure to ethanol induces the activation of microglia, which subsequently leads to the release of inflammatory mediators. This activation also initiates intracellular signaling pathways that contribute to the synthesis of pro-inflammatory cytokines (such as IL-1β and TNF-α) and enzymes (including COX-2 and iNOS) involved in the inflammatory response [23,24,25]. To examine the impacts of co-administration of ACR and IAE on neuronal inflammation, we evaluated NF-κB, TNF-α, and PGE2 levels in the brain using rat ELISA kits. In our study, TNF-α levels showed an increase in all treatment groups related to the control rats, with no significant difference between the ACR + IAE group and the ACR-alone group or the IAE-alone group. However, reduced levels of PGE2 were observed in both the ACR group and the ACR + IAE group. In particular, the ACR + IAE group exhibited a significant difference when compared to the IAE-alone group. Similarly, the ACR group exhibited a significant difference compared to the IAE-alone group. Additionally, there was no noteworthy distinction observed between the ACR + IAE group and the ACR-alone group. In terms of NF-κB, as anticipated, the co-administration of ACR and IAE led to the most substantial increase in levels among all treatment groups, including the normal group. All groups receiving treatment exhibited a notable rise in NF-κB levels compared to the control rats.

Apoptotic cell death has been linked to oxidative stress in neurodegenerative disorders. The heightened production of ROS within neurons disturbs mitochondrial function, initiating a detrimental cycle of ROS-induced damage. Eventually, this process causes apoptosis in neuronal cells. In the intrinsic pathway of apoptosis, the pro-apoptotic protein Bax plays a role by releasing Cytochrome c from mitochondria in response to external pro-apoptotic signals. The release of Cytochrome c then activates Caspase-3 by cleaving Procaspase-3. The activated Caspase-3 initiates cellular death by causing degradation of cellular components and the occurrence of DNA damage. To counteract this mechanism, the anti-apoptotic protein Bcl-2 inhibits the Bax-mediated release of Cytochrome c from the mitochondria. In the extrinsic pathway of apoptosis, TNF-α binds to death receptors located on the cell membrane, initiating the activation of Caspase-8. As a result, Caspase-8 activates Caspase-3 from Procaspase-3, leading to cell death. Research on ACR found that it induced mitochondrial dysfunction in human astrocytoma cells and BV-2 microglia [57,58]. ACR further stimulated Caspase-9 and its downstream signaling cascade, enhanced the Bax/Bcl-2 ratio, and induced apoptosis and neurotoxicity via mitochondrial-dependent mechanisms [57]. Ethanol exposure leads to notable apoptotic effects on purkinje cells in the developing cerebellum [59]. Additionally, it plays a direct role in generating ROS and triggering apoptosis. Moreover, in a dose-dependent manner, it heightens lead-induced oxidative stress and initiates apoptosis in neuronal cells [60,61,62]. In this study, as anticipated, the levels of Bcl-2 demonstrated a significant decrease in all treatment groups compared to the normal group. Moreover, no notable distinction was detected among the ACR + IAE group and the ACR-alone group, or the IAE-alone group. Regarding Bax levels, no notable distinction was detected among the ACR + IAE group and the ACR-alone group. However, a noteworthy difference was observed in the IAE-alone group, as Bax levels exhibited a significant increase in the ACR + IAE and ACR groups. Additionally, the levels of Caspase-3 showed a significant increase in all treatment groups compared to the normal group. Nevertheless, there were no noteworthy variations observed between the ACR + IAE group and the ACR-alone group. Significantly, both the ACR + IAE group and the ACR-alone group exhibited a more notable reduction compared to the IAE-alone group.

Oxidative stress pertains to a disturbance in the equilibrium of oxidants and antioxidants in an organism. This imbalance can occur due to an excessive presence of ROS or impaired functioning of the antioxidant system [63]. In this study, we assessed oxidative stress levels by measuring various parameters related to oxidation and antioxidation, including MDA, CAT, and GSH. These parameters were selected based on their specific roles and characteristics. MDA serves as a marker of oxidative stress, formed during lipid peroxidation and elevated in patients with epilepsy, indicating increased oxidative stress [64]. CAT, on the other hand, acts as a potent antioxidant enzyme with a hemoprotein structure containing four heme groups, converting hydrogen peroxide into water and oxygen [65]. GSH plays a vital role in eliminating ROS within cells and exists in two forms: endogenous, produced internally, and exogenous, obtained from external sources [66]. Through covalent adduct formation with cysteine residues in presynaptic neurons, ACR disrupts neurotransmitter transfer and leads to neurotoxic effects. Oxidative stress also contributes to ACR-induced neurotoxicity, acting as a biochemical and physiological signaling mechanism [15,17,18]. Additionally, chronic alcohol consumption disrupts the balance between free radical formation and the body’s antioxidant defenses, leading to elevated oxidative stress. This imbalance leads to lipid peroxidation, protein inactivation, DNA damage, impaired mitochondrial function, cell death, and heightened inflammatory response [67]. In terms of our assessment of oxidative and antioxidative parameters, all treatment groups (ACR, IAE, and ACR + IAE) demonstrated changes in MDA, GSH, and CAT levels compared to the normal group. Nevertheless, with the exception of a significant rise in MDA levels in the ACR + IAE group in comparison to the ACR group, there were no significant variances noted between the treatment groups.

A photomicrograph of the rat brain tissue demonstrated that there were alterations in morphological changes in all treatment groups: ACR, IAE, and ACR + IAE. The IAE group showed a very mild form of red neurons and gliosis, which may be because of intermittent alcohol exposure and a low dose. The histopathological features of the ACR group showed red neurons as well as reactive gliosis. In the present study, the histopathological features of the ACR + IAE group exhibited more aggression, with the presence of red neurons and reactive gliosis being more noticeable than in the ACR group. These results suggest that the ACR + IAE group experiences more extensive brain injury than the ACR group. In contrast to all treatment groups, the control group shows unremarkable morphological changes. Additionally, a previous study with the same dose and duration of ACR exposure as our experiment demonstrated alterations in the histopathological features of the brain cortex, including lesions such as necrosis of neurons and glial cells [34]. Moreover, a previous study also demonstrated the occurrence of reactive astrogliosis following a short-term episode (one or two days) of binge 25% ethanol exposure [68]. In previous studies, ethanol exposure was very brief (one or two days) and involved 25% ethanol. Our study, however, examines the effects of IAE exposure of ethanol and the combined effects of ACR + IAE.

Collectively, the tobacco products appear to be the second most significant source of ACR exposure for people, after dietary intake from food [15]. As mentioned earlier, cigarette smoke contains between 0.5 and 4.3 micrograms of ACR [10]. Therefore, our research aimed to provide preliminary insights into the effects of tobacco abuse in conjunction with oral ACR exposure. Moreover, the concurrent consumption of tobacco and ethanol is common among abusers. Our present results provide evidence of the neurotoxic effects of acrylamide from combined tobacco and ethanol consumption.

## 5. Limitations

The present study represents a preliminary approach targeting a number of related mechanisms and needs to be extended to the molecular level for a clearer understanding in the future. Although the study included several mechanisms, the markers for each were limited. In particular, for neuroinflammation, while pro-inflammatory markers were covered, anti-inflammatory markers were not adequately represented. Most results showed significant differences compared to control rats, but clarity was lacking between the treatment groups, despite notable significant differences between the individual treatments and the combined administration. Extending the administration period might be necessary to observe significant changes. Regarding histopathology, the number of glial cells in different brain regions is typically variable. Consequently, there is no well-established cutoff point that distinguishes between the normal number of glial cells and gliosis. This variability posed a limitation in using the number of glial cells to support neuronal toxicity in this study.

## 6. Conclusions

During the administration of ACR, IAE, and their combination continuously for 30 days, changes were observed in TL values in the EPM test on days 26–27, exploration time and discrimination capability between FO and NO in the NOR test on days 28–29, and the number of entries and duration of time spent in the novel arm in the Y-maze test on day 30 compared to the control group. However, there were no significant differences found between the treatments. Additionally, the treatments induced neuroinflammation, oxidative stress, and apoptosis in the brain, with most parameters being similar between the ACR and ACR + IAE treatments. Histopathological features revealed significant changes in ACR + IAE brain structures as compared to the ACR- and IAE-alone groups. Further research is required to explore the long-term effects of co-administering ACR and IAE on additional behavioral models and molecular mechanisms to elucidate the impact of combining these administrations on brain toxicity. Moreover, more extensive research is necessary to investigate the impact of alcohol and ACR metabolism when co-administered, as well as to provide further evidence regarding their combined abuse.

## Data Availability

The data presented in this study are available in the article.
